# Sources of solutes and carbon cycling in perennially ice-covered Lake Untersee, Antarctica

**DOI:** 10.1038/s41598-020-69116-6

**Published:** 2020-07-23

**Authors:** Nicole B. Marsh, Denis Lacelle, Benoit Faucher, Sarina Cotroneo, Liam Jasperse, Ian D. Clark, Dale T. Andersen

**Affiliations:** 1 Department of Earth and Environmental Science, University of Ottawa, Ottawa, ON Canada; 2 Department of Geography, Environment and Geomatics, University of Ottawa, Ottawa, ON Canada; 3 Carl Sagan Center, SETI Institute, Mountain View, CA USA

**Keywords:** Limnology, Cryospheric science, Biogeochemistry

## Abstract

Perennially ice-covered lakes that host benthic microbial ecosystems are present in many regions of Antarctica. Lake Untersee is an ultra-oligotrophic lake that is substantially different from any other lakes on the continent as it does not develop a seasonal moat and therefore shares similarities to sub-glacial lakes where they are sealed to the atmosphere. Here, we determine the source of major solutes and carbon to Lake Untersee, evaluate the carbon cycling and assess the metabolic functioning of microbial mats using an isotope geochemistry approach. The findings suggest that the glacial meltwater recharging the closed-basin and well-sealed Lake Untersee largely determines the major solute chemistry of the oxic water column with plagioclase and alumino-silicate weathering contributing < 5% of the Ca^2+^–Na^+^ solutes to the lake. The TIC concentration in the lake is very low and is sourced from melting of glacial ice and direct release of occluded CO_2_ gases into the water column. The comparison of δ^13^C_TIC_ of the oxic lake waters with the δ^13^C in the top microbial mat layer show no fractionation due to non-discriminating photosynthetic fixation of HCO_3_^–^ in the high pH and carbon-starved water. The ^14^C results indicate that phototrophs are also fixing respired CO_2_ from heterotrophic metabolism of the underlying microbial mats layers. The findings provide insights into the development of collaboration in carbon partitioning within the microbial mats to support their growth in a carbon-starved ecosystem.

## Introduction

Numerous perennially ice-covered lakes have been inventoried in Antarctica, including in the McMurdo Dry Valleys (MDV), Bunger Hills, Vestfold Hills, Schirmacher Oasis, and Soya Coast^1,2^. These lakes have varied chemistries as a result of source water and Holocene history of the lakes, but many are oligotrophic and support benthic cyanobacterial mats and heterotrophic bacterial communities^3–5^. Primary productivity is often limited by light attenuation through the ice-cover and nutrient availability (e.g., C and P), however, summer moating and streams provide seasonal recharge of nutrients to the lakes^4,6^. Analysis of carbon isotopes (e.g., δ^13^C, ^14^C) can provide insights about the source of carbon and transformations of organic matter as photosynthesis and remineralization control the isotopic composition of most organic matter. For example, carbon isotope geochemistry of dissolved inorganic carbon (δ^13^C_DIC_) and organic carbon (δ^13^C_DOC_) have been used to trace carbon sources and cycling in the MDV lake ecosystem^3,7–9^.

Lake Untersee is a 169-m deep ultra-oligotrophic lake that is substantially different from other lakes in Antarctica^10,11^. It is recharged by subaqueous melting of glacial ice and subglacial meltwater, and the lake remains ice-covered with no open water along the margin (summer moating) that would provide access to nutrients, CO_2_ and enhanced sunlight^12–14^. In the absence of large metazoans, photosynthetic microbial mats cover the floor of the lake from just below the ice cover to depths exceeding 130 m, including the formation of small cupsate pinnacles and large conical structures growing in a light and nutrients-starved waters; to date, Untersee is the only freshwater lake hosting the formation of modern large conical stromatolites^12,15^. Metagenomic sequencing of the 16S rRNA gene showed that the top layer of the microbial mats is composed of a community of carbon-fixing cyanobacteria (Phormidium sp., Leptolyngbya sp., and Pseudanabaena sp) that shifts to a heterotrophic community in the underlying layers (Actinobacteria, Verrucomicrobia, Proteobacteria, and Bacteroidetes) with amino acid-, carbohydrate-, and arsenic-predicted metabolisms^16^. The photosynthetic mats are active but with much lower gross photosynthesis and sequestration rates of organic carbon than mats in MDV lakes^15^.

Previous work at Lake Untersee characterized the geochemistry of the water column^10,11,17^ and examined the structure of the microbial communities^16^. However, studies have yet to explore the effect of the absence of summer moating on weathering, the carbon sources and carbon cycling in the lake ecosystem. Here, we assess the source of major solutes and carbon to Lake Untersee, carbon cycling and functioning of the microbial ecosystem using an isotope geochemistry approach. This objective was accomplished by determining the concentration of major ions, δ^34^S_SO4_ and Sr-isotopes, total inorganic carbon (TIC), total organic carbon (TOC), δ^13^C and ^14^C of the TIC and TOC in the lake water column and the abundance of organic carbon, δ^13^C, and ^14^C in the microbial mats. The results are compared to existing biogeochemistry datasets of firn cores from Dronning Maud Land and ice-covered lakes in the McMurdo Dry Valleys that develop summer moats. The findings have relevance to ice-covered lakes on early Mars and ice-covered oceans of icy moons such as Enceladus^18^.

### Study area

**Figure 1:**
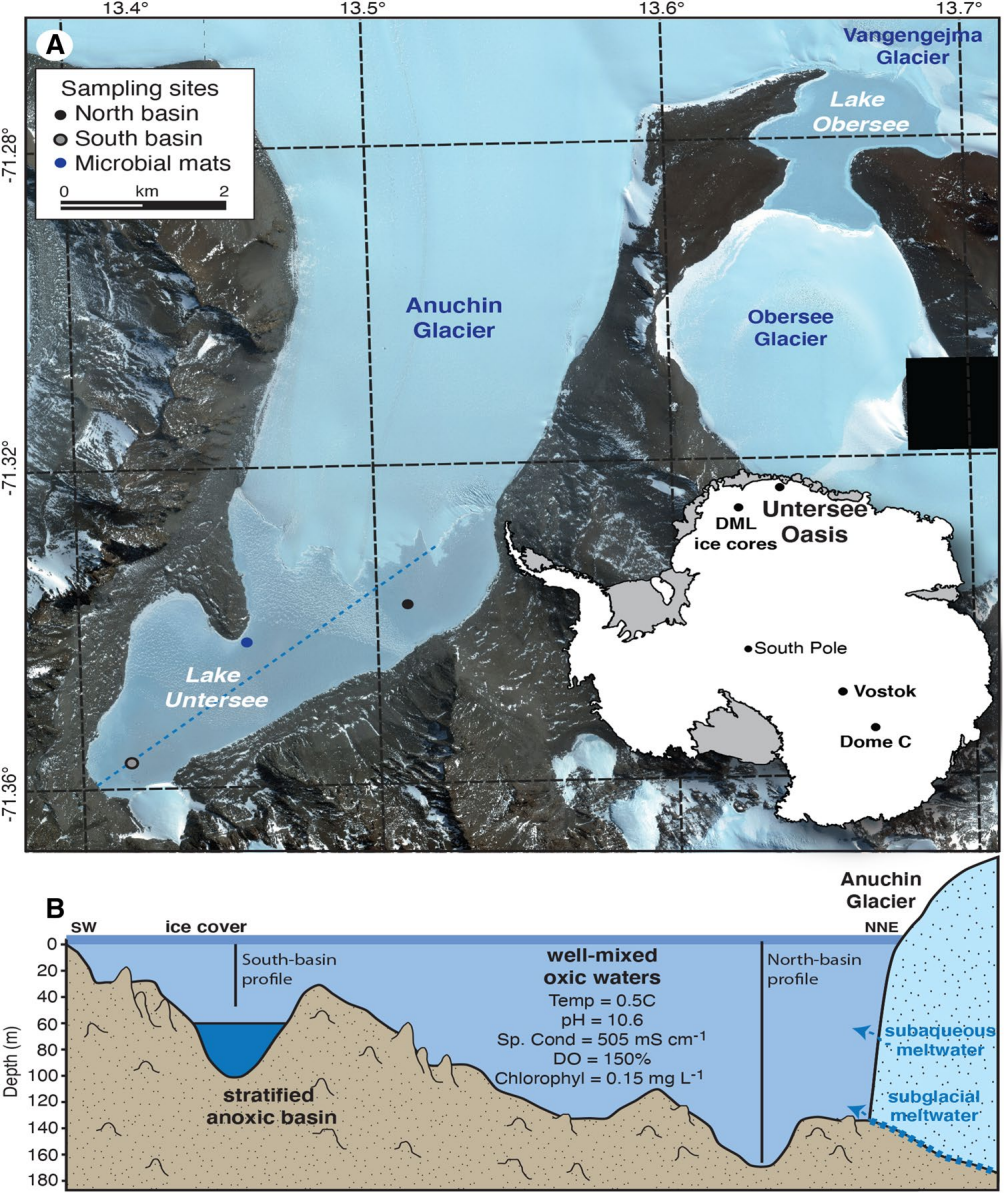
(**A**) Location map of Lake Untersee in central Dronning Maud Land, Antarctica. (**B**) Cross-section of bathymetry of Lake Untersee and location of sampling in north and south basins. Background Digital Globe satellite imagery, December 7, 2017; ©2020 Digital Globe NextView License (provided by NGA commercial imagery program). Map generated using ArcGISv10.

Lake Untersee (71° 20.736′ S; 13° 27.842′ E) is located in the Untersee Oasis in a steep-sided valley in the Gruber Mountains, approximately 90 km southeast of the Schirmacher Oasis and 150 km from the coast^19^ (Fig. 1). The local geology consists of norite, anorthosite and anorthosite-norite alternation of the Precambrian Eliseev anorthosite complex^20^. Lake Untersee developed at 12–10 ka BP and measures 2.5 km wide and 6.5 km long, making it the largest freshwater lake in central Dronning Maud Land (DML)^10^. Untersee Oasis is part of a polar desert regime, the climate subjecting the area to intense ablation limits surface melt features due to cooling associated with latent heat of sublimation (e.g., Refs.^13,21, 22^).

Lake Untersee has two sub-basins; the largest, 160 m deep, lies adjacent to the Anuchin Glacier and is separated by a sill at 50 m depth from a smaller, 100 m deep basin to the south. The northern deep basin is well-mixed due to buoyancy-driven convection caused by melting of the ice-wall at the glacier-lake interface^11,23^; however, the smaller southern basin is chemically stratified below the sill depth (c. 50 m) and its higher density prevents mixing with the overlying oxic water column^24,25^. Lake Untersee loses c. 1% of its water annually from the sublimation of the 2–4 m thick ice cover. To maintain hydrological balance the lake must be recharged by an equal inflow (i.e., Refs.^13,26^). The lake is dammed at its northern sector by the Anuchin Glacier and mass balance calculations suggest that subaqueous melting of terminus ice contributes 40–45% of the annual water budget with subglacial meltwater contributing the remainder^13^. Based on δD-δ^18^O of the water column, the lake has not developed a moat for at least the past 300–500 years^17^. The oxic water column has a Na(Ca)–SO_4_ geochemical facies with uniform temperature (0.5 °C), pH (10.5), dissolved oxygen (c. 150%), specific conductivity (c. 505 µS cm^–1^) and chlorophyll (0.15 µg L^−1^)^11,12^. The nutrients are very low with TIC (0.14 mg C L^−1^), NO_3_ (0.2 mg L^−1^), NO_2_ (0.11 mg L^−1^), NH_4_ (40 µg L^−1^) and phosphate (< 1 µg L^−1^)^15,27^. Approximately 5% of incident irradiance is transmitted through the lake ice, with a vertical extinction coefficient for scalar photosynthetically active radiation (PAR) of 0.033 m^−1^, resulting in a 0.1% surface irradiance depth of ~ 135 m^12^. Lake Untersee supports an exclusively microbial ecosystem along its lake bed with photosynthetic microbial mat communities observed to depths of at least 130 m^12,15^. The mats are composed of filamentous cyanophytes that form mats, cm-scale cuspate pinnacles, and large conical stromatolites that rise up to 70 cm above the lake floor. The mats are characterized by lamina of organic material and silt- and clay-sized sediments with occasional blocky plagioclase crystals. The fine-grained sediment enters the lake as glacial flour through subglacial melting and deposited on the microbial mats.

## Results and discussion

### Source of major solutes to Lake Untersee

**Figure 2:**
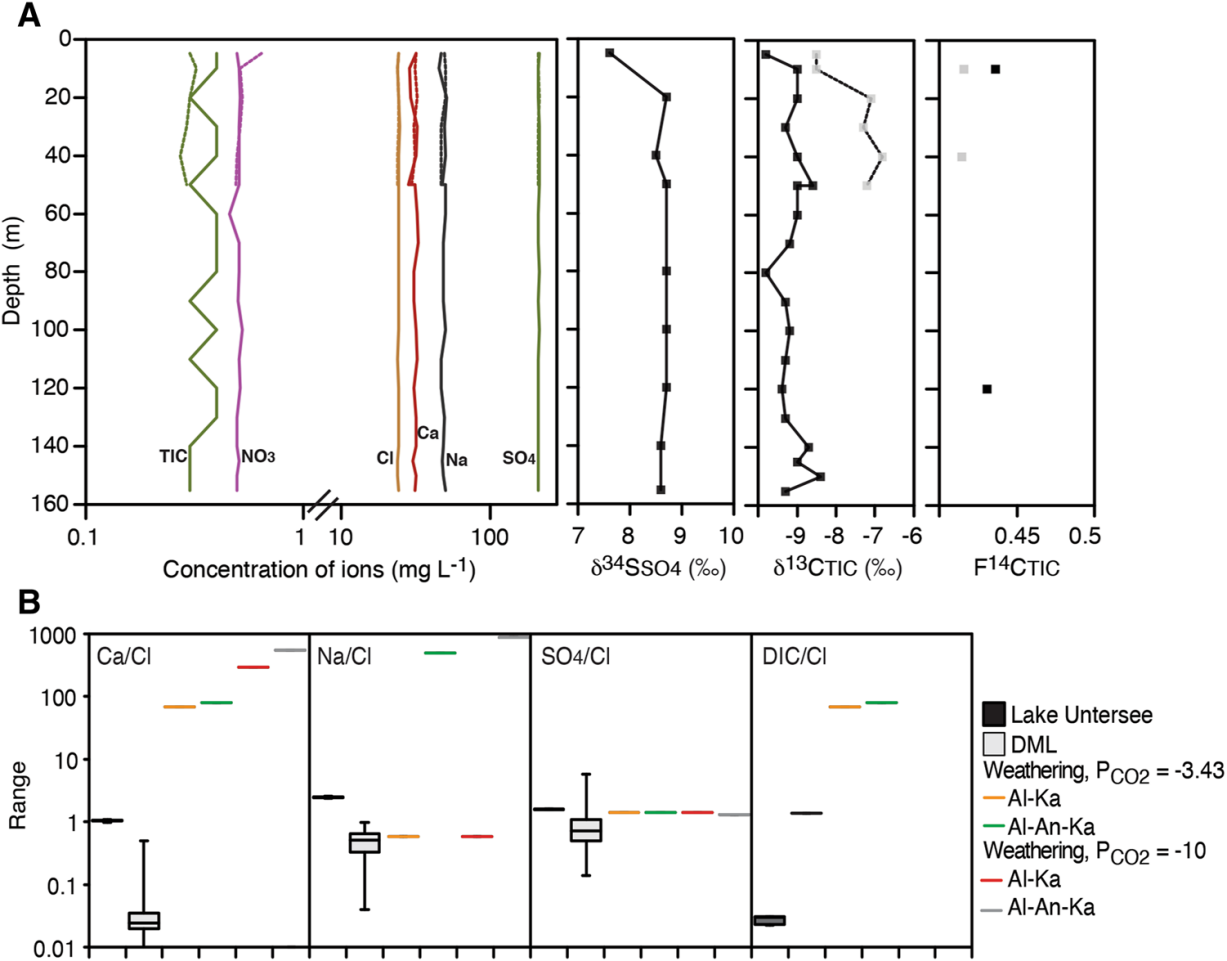
(**A**) Geochemical profiles in North and South basins of Lake Untersee, Antarctica. Ionic content, δ^34^S_SO4_, δ^13^C_TIC_ and F^14^C_TIC_. (**B**) Molar ratios (Ca/Cl, Na/Cl, SO_4_/Cl and DIC/Cl) in Lake Untersee compared to DML Core Epica and weathering of plagioclase minerals. Weathering simulation were performed with PHREEQC hydrogeochemical modeling. See Table 2 for description. *Al* albite, *An* anorthite, *Ka* kaolinite.

Lake Untersee has a Na(Ca)–SO_4_ geochemical facies with [SO_4_^2−^], [Na^+^] and [Ca^2+^] of 166 ± 0.7, 61.8 ± 1.2, 45.7 ± 1.2 mg L^−1^, respectively (Fig. 2). The δ^34^S_SO4_ values range from 7.6 to 8.7‰. The water column is also characterized by Sr^+^ (18 ± 0.2 µg L^−1^) with very radiogenic ^87^Sr/^86^Sr ratios (0.71804–0.71832; Table 1). To gain insight into the source of solutes to Lake Untersee, the major solutes and their isotopes are first compared to firn cores from the DML region. The nearby Core Epica in western DML is characterized by a similar Na-SO_4_ facies but with solute concentration 3–4 orders of magnitude lower than in the lake^28^. The water column in Lake Untersee undergoes cryo-concentration of solutes as water is frozen onto the bottom of the ice cover and the solutes remain mostly in the water column (i.e., Refs.^29,30^). The effective segregation coefficient (Keff) during freezing varies between solutes as it is dependent on solubility, molecular symmetry and the structure of the crystal lattice^29^. A recent study of lake ice formation calculated from their freezing experiments that the segregation coefficient is similar for Ca-Na-Cl (Keff: 0.133–0.177), however, it is much higher for SO_4_ (Keff: 0.299 ± 0.025) indicating that more SO_4_ is incorporated in the ice^30^. If we first correct the solute concentration for ionic segregation during freezing using the Keff for respective solutes, the effect of solute enrichment by cryo-concentration can then be removed by normalizing the solutes to Cl^−^, a conservative tracer.

**Table 1:** Strontium isotopes in Lake Untersee and Precambrian Eliseev anorthosite complex in the Untersee Oasis (Antarctica).

Sample	Sample Type	^87^Sr/^86^Sr (± 2σ)	References
**Lake Untersee**			
North basin 10 m depth	Water	0.71831 (0.000164)	This study
North basin 80 m depth	Water	0.71830 (0.000184)	This study
North basin 155 m depth	Water	0.71829 (0.000228)	This study
South basin 10 m depth	Water	0.71832 (0.000180)	This study
**Modern seawater**	Water	0.70918	1
**Eliseev Anorthosite Complex, Untersee Oasis**			
W74 Norite	Rock	0.7119	2
W20 Noritic Anorthosite	Rock	0.7091	2
W94 Anorthosite	Rock	0.7079	2
W100–1 Anorthosite	Rock	0.7107	2
W100–2 Anorthositic gabbro	Rock	0.7102	2
W100–3 Anorthosite	Rock	0.7085	2
**McMurdo Dry Valleys lakes and streams**			
Taylor Valley	Water	0.70894–0.71333	3
Wright Valley	Water	0.7144–0.7185	4
**Antarctica Ice Core Data**			
Dome C dust (18 ky)	Dust	0.708707	5
Dome C ice (7.5–23 ky)	Ice	0.7068–0.7097	6
Law Dome EH (65 ky)	Ice	0.0793–0.7097	6
Vostok ice	Dust	0.708047–0.711254	1
Vostok type 1 accretion ice	Ice	0.71655	1

Also shown for comparison is Sr isotopes from seawater, lakes and streams in the McMurdo Dry Valleys and Antarctic ice cores.

Refs. 1. Lyons et al. (2016)^67^; 2. Bormann and Fritzsche (1995)^19^; 3. Lyons et al. (2002)^68^ 4. Friedman et al. (1995)^69^ 5. Grousset et al. (1992)^70^; 6. Burton et al. (2002)^71^.

Lake Untersee has significantly higher SO_4_/Cl ratios (1.765 ± 0.002) compared to those in Core Epica (0.89 ± 0.61) (one-way ANOVA, F = 25.88; *p* < 0.001). The lake also has significantly lower δ^34^S_SO4_ (7.6 to 8.7‰) than the δ^34^S_tot_ values from nearby CV and CM (14.5–15‰; Ref.^31^). The difference in SO_4_/Cl ratios and δ^34^S_SO4_ indicates the input of SO_4_ to Lake Untersee is c. 50% higher with a depleted δ^34^S composition relative to the firn cores from western DML. The western DML cores are situated 400–600 km from the Untersee Oasis and span only the past 50–1,500 years, whereas Lake Untersee has been accumulating solutes since its formation c. 12–10 ka years ago. Despite these spatio-temporal differences in the SO_4_ data, and in the absence of a deep ice core with SO_4_ and δ^34^S measurements from DML, inferences on possible SO_4_ sources to Lake Untersee can still be made. First, a local source of SO_4_ can be ruled out since sulfate evaporites are not found in the vicinity of the Untersee Oasis. Secondly, volcanic eruptions are identifiable as distinct SO_4_ peaks in ice cores and have δ^34^S values in the 0 to 5‰ range^32^. Core Epica shows five SO_4_ volcanic peaks between 1932 and 1991 (representing c. 2% of total SO_4_ in the core); however, the EDC96 SO_4_ record shows that volcanic SO_4_ contributed ~ 6% of the total SO_4_ during the Holocene^33^. Finally, the concentration of SO_4_ and δ^34^S_SO4_ from Dome C and Taylor Dome ice cores are 2–3× higher and c. 2‰ lower (9.5–12.6‰), respectively, during the last glacial maximum than during the Holocene as a result of increased meridional transport of marine biogenic SO_4_ and terrestrial dust^34–36^. Therefore, despite the differences in SO_4_/Cl and δ^34^S_SO4_ between Lake Untersee and the nearby DML firn cores, it appears that SO_4_ in the lake is sourced from glacial meltwater. Considering that Lake Untersee formed c. 12–10 ka years ago during deglaciation, the initial late Pleistocene glacial meltwater filling the basin would likely have had a higher SO_4_/Cl with depleted δ^34^S signature relative to shallow firn cores from western DML. Then, continuous recharge from glacial meltwater during the Holocene with likely higher volcanic SO_4_ and SO_4_-bearing terrestrial dust load (both with low δ^34^S; i.e., Refs.^32,37^), as evidenced from the EDC96 SO_4_ record, also contributed to the SO_4_ in Lake Untersee.

Lake Untersee also has significantly higher Na/Cl and Ca/Cl molar ratios (2.468 ± 0.0483 and 1.048 ± 0.027, respectively) compared to those in Core Epica (0.494 ± 0.213 and 0.0336 ± 0.0365, respectively (one-way ANOVA, Na/Cl: F = 7,013.3, *p* < 0.001; Ca/Cl: F = 770.2, *p* < 0.001) (Fig. 2). The higher ratios can be explained in part to the higher [Na^+^] and [Ca^2+^] in glacial ice during last glacial maximum^34^, however the Na/Cl at Dome C only averages 1.83 ± 1.80 over the past 44 ka^34^. References^10,27^ suggested that the enrichment in Na^+^ and Ca^2+^ can be attributed to weathering of silicate minerals (i.e., plagioclase: albite, anorthite and kaolinite). X-ray diffraction analysis revealed that the mineralogy of the fine particles in the mats includes anorthite (40–58%) and kaolinite (7–10%), with a few discrete sand layers having higher quart/tremolite. Here, we test the effect of plagioclase weathering in the water column under closed-system condition, which simulates a well-sealed lake with no exchange of gases. Geochemical modeling using PHREEQC hydrogeochemical software^38^ predicts that closed-system (*P*_CO2_ = –10) weathering of plagioclase minerals from glacial meltwater would generate water with a pH of 7.7–11.7 with a range of Na/Cl and Ca/Cl ratios that depend on the mineral assemblage: in all modeled scenarios Ca/Cl ratios (18 to 542) were higher than values in Lake Untersee; however only weathering of the mineral assemblage with albite yielded Na/Cl ratios higher than the lake (Table 2). Therefore, a two-component mixing calculation between the modeled Ca/Cl and Na/Cl ratios and glacial meltwater composition from Core E suggests that weathering of plagioclase contributes a minor portion of the Na^+^ and Ca^2+^ load (< 5%) relative to solutes contributed from glacial melt.

**Table 2:** Results of numerical simulations using PHREEQC hydrogeochemical software^38^.

	pH	DIC (ppm C)	Ca (ppm)	Na (ppm)	SO_4_ (ppm)	Cl (ppm)	Ca/Cl	Na/Cl	SO_4_/Cl	DIC/Cl
**Glacial water**	5.6	0.00165	1.20E−03	1.35E−02	6.15E−02	3.56E−02	0.030	0.58	0.64	0.14
**Eq. w/atm CO**_**2**_	5.4	0.37	1.20E−03	1.35E−02	6.16E−02	3.56E−02	0.030	0.59	0.64	30.5
**Open system weathering (P**_**CO2**_** = **−**3.43)**										
al, an	7.0	1.20	1.05	1.06	0.06	0.036	26.0	45.8	0.64	99.5
al, an, ka	10.3	1.51	5.02	17.51	0.09	0.056	79.8	485.4	0.63	79.9
an, ka	10.5	1.29	4.30	0.02	0.09	0.056	68.3	0.58	0.63	68.5
an, ka, pl	10.5	1.30	4.31	0.00	0.09	0.056	68.5	5.0E−16	0.63	69.2
**Closed-system weathering (P**_**CO2**_** = **−**10)**										
al, an	7.7	0.00	0.73	1.06	0.06	0.036	18.2	45.89	0.64	0.00
al, an, ka	11.6	0.11	34.11	31.66	0.09	0.056	541.7	876.5	0.59	5.70
an, ka	11.7	0.16	18.35	0.02	0.09	0.056	291.4	0.58	0.61	8.59
an, ka, pl	11.7	0.16	18.36	0.00	0.09	0.056	291.6	2.5E−19	0.61	8.59

Simulations were conducted using as input the average chemical composition of glacial meltwater (Core Epica, western Dronning Maud Land^28^) and were equilibrated with atmospheric CO_2_ (*P*_CO2_ = –3.43) or we simulated weathering of plagioclase mineral under closed-system (*P*_CO2_ = –10), which simulates a well-sealed lake with no exchange of gases. Calcite is allowed to precipitate if saturation is reached.

*al* albite, *an* anorthite, *ka* kaolinite, *pl* plagioclase.

Strontium in Lake Untersee is considerably more radiogenic (0.71804–0.71832) than the local anorthosite and norite (0.7079–0.7118), dust in glacial ice (0.708047–0.711254) and modern sea-water (0.70918) (Table 1). Therefore, a more radiogenic source of Sr is needed to explain the Sr isotope ratios in the lake. Refs.^39,40^ demonstrated the preferential solubilization of ^87^Sr in the early stages of weathering of glacial deposits due to the rapid weathering of biotite relative to plagioclase. Although the regional basement rocks are dominantly anorthosite, norites and some dykes, the morainic material contains boulders of varied lithology including biotite-bearing gneisses. Thus, weathering products from glacial scouring and/or subglacial weathering of the biotite-bearing gneiss may enrich the ^87^Sr/^86^Sr in the lake waters with respect to the signature in the local Eliseev anorthosite complex.

The hydrological conditions of Lake Untersee play a key role on determining the major solute chemistry of the oxic water column. The closed-basin lake loses water only by sublimation of its ice cover and is recharged by subaqueous melting of glacial ice and subglacial meltwater with no summer moating^13^. The major solutes in Lake Untersee reflect a heritage of 12–10 ka of recharge from glacial meltwater and cryo-concentration of the ionic load in the water column. Early in its existence, the lake would have received a higher ionic concentration from late Pleistocene glacial meltwater that shifted to lower ionic load from glacial meltwater during the Holocene (i.e., Refs.^34–36^). Weathering of local plagioclase contributes a small fraction of ions and Sr isotopes suggest weathering from biotite-bearing gneisses within the drainage basin. However, it is still unclear if weathering occurs primarily while glacial flour is suspended in the lake, within the mats on the lake floor where a higher *p*CO_2_ from respiration would enhance weathering, or in the subglacial meltwaters along the basement rocks.

### Source, fixation and cycling of carbon in Lake Untersee

The oxic water of Lake Untersee has TIC concentration near detection limit (0.3–0.4 mg C L^−1^) with δ^13^C_TIC_ ranging from –6.8 to –9.8‰ (average = –9.1 ± 0.4‰) (Fig. 2). The measured TIC concentration are similar to those measured by Ref.^15^ (0.14 mg C L^−1^), but significantly lower than values reported by Ref.^25^ (DIC: 2–8 mg C L^–1^; δ^13^C_DIC_: 4.3‰). The higher DIC and δ^13^C_DIC_ measured by Ref.^25^ is likely due to atmospheric contamination during sampling and filtering of samples in the field. When exposed to air during filtering, the poorly-buffered high pH lake water (pH 10.5) quickly exchanges with atmospheric CO_2_; PHREEQC modeling calculates DIC of 1.5 mg C L^−1^ and δ^13^C_DIC_ 3‰ for lake water equilibrating with atmospheric CO_2_. The TOC in the oxic water was below the detection limit (< 0.3 mg C L^–1^) in most samples (one sample at 40 m depth in the south basin yielded DOC of 0.4 mg C L^−1^ with δ^13^C_DOC_ of – 27.5‰). However, two composite 1L samples analyzed for ^14^C_TOC_ yielded 0.27 mg C and 0.22 mg C, suggesting that TOC is c. 0.12 mg C L^–1^. The TOC is about one order of magnitude lower than in other perennially ice-covered Antarctic surface lakes (c. 2.4 mg C L^−1^; Ref.^41^), but is closer to the average compiled for Antarctic glacial ice determined from a range of ice sheet and valley glaciers (0.14 mg C L^−1^; Ref.^42^). Tritium (^3^H) and radio-iodine (^129^I) measurements were below detection limit in the water column (< 0.8 TU and < 7.5 × 10^5^ atoms L^–1^, respectively). Radiocarbon analysis of TIC yielded similar F^14^C_TIC_ (0.414 to 0.436), which is equivalent to apparent ages of 6,666 ± 98 to 7,054 ± 88 years BP (Table 3). For ^14^C_TOC_, two pairs of samples were combined to obtain sufficient carbon for analysis (NB-10 and NB-40; NB-60 and NB-120) and yielded F^14^C_TOC_ of 0.42 ± 0.02 (6,906 ± 610 years BP) and 0.55 ± 0.02 (4,734 ± 606 years BP), respectively (Table 3).

**Table 3:** Radiocarbon results of total inorganic carbon (TIC) and total organic carbon (TOC) in Lake Untersee, Antarctica.

Sample ID	Depth (m)	F^14^C_TIC_	^14^C year BP (± 2σ)	Lab ID
**North Basin, 2017**				
NB-10	10	0.4361 (0.0054)	6,666 (98)	UOC-6214
NB-40	40	0.5989 (0.0132)	4,119 (176)	UOC-6215
NB-120	120	0.4307 (0.004)	6,766 (76)	UOC-6217
**South Basin, 2017**				
SB-10	10	0.4156 (0.0046)	7,054 (88)	UOC-6219
SB-40	40	0.4143 (0.0038)	7,079 (76)	UOC-6220

		F^14^C_TOC_	^14^C year BP (± 2σ)	
**North Basin, 2017**				
NB-10–40	10–40	0.4233 (0.0322)a	6,906 (610)a	UOC-6540
NB-80–120	80–120	0.5547 (0.0418)b	4,734 (610)b	UOC-6541

F^14^C, fraction modern carbon; ^14^C year BP, years before 1950; a, samples NB-10 and NB-40; b, samples NB-80 and NB-120 were combined to yield sufficient carbon for analysis.

The TIC (0.35 mg C L^−1^) and δ^13^C_TIC_ (– 9.1 ± 0.4‰) in Lake Untersee are first compared to six oligotrophic closed-basin lakes in the MDV. The wide range in DIC (2 to 1,000 mg C L^−1^) and δ^13^C_DIC_ values (– 5 to 10‰) of the MDV lakes are a product of current and past recharge mechanisms, conditions of the ice cover, and biogeochemical processes occurring in the lakes, including mineralization of organic matter and dissolution of CaCO_3_. The DIC is brought into the MDV lakes by ephemeral proglacial streams and its concentration is largely influenced by ice cover phenology: DIC is higher in lakes that did not lose their ice cover over the past few millennia and the effect of cryo-concentration (i.e., Bonney, Vanda, Fryxell); DIC is lower in lakes that lost their ice cover due to CO_2_ degassing and calcite precipitation as the pH decreased (i.e., Hoare, Joyce; Refs.^3,8,43^). The δ^13^C_DIC_ of the MDV lakes also shows that DIC is not a limiting factor to primary productivity of photosynthetic mats. When abundant CO_2_ is available for carbon fixation by the Rubisco enzyme, photosynthetic activity preferentially utilizes ^12^C, producing microbial mats with depleted δ^13^C and leaving the residual DIC enriched in ^13^C^7,44,45^. The maximum fractionation factor by cyanobacteria ranges from 1.023 to 1.018^46,47^, which is the difference in δ^13^C_DIC_ and δ^13^Cmats in many MDV lakes^7,45,48^. Conversely, re-mineralization of δ^13^C depleted organic matter would produce δ^13^C_DIC_ with similarly low values, as seen near the bottom of Lake Vanda^7^. Based on the findings of MDV lakes, a low TIC in Lake Untersee is unexpected considering that Untersee has been well-sealed for the past few hundred years^17^ and likely throughout most of the history of the lake due to the paucity of calcite in the mats^12^. The mats in Lake Untersee are active, albeit with a lower gross photosynthesis and sequestration rates of organic carbon than mats in MDV lakes^15^, and as such the δ^13^C_DIC_ would be expected to be similar to those in the MDV lakes. A significant question then is, why are the TIC and δ^13^C_TIC_ in Lake Untersee so different from those in the MDV lakes?

The much lower DIC concentration in Lake Untersee can be attributed to the absence of ephemeral surface streams feeding the lake and its rather unique recharge mechanism: glacial meltwater that does not come in direct contact with the atmosphere. The subaqueous melting of the Anuchin Glacier releases occluded air directly into the water column and the occluded atmospheric CO_2_ gas in the ice then becomes a contributor to the TIC pool. Concentrations of TIC and δ^13^C_TIC_ in the subaqueous meltwater can be estimated from the occluded gas volume and its age, firn porosity at the depth of closure and CO_2_ concentration. The Anuchin Glacier at the lake-glacier interface is c. 160 m thick and likely contains occluded gases with an average age of 1,800 years (i.e., based on age-depth relation of occluded gases from nearby EDML ice core;^17^). Assuming an average gas volume of 0.11 cm^3^ g^–1^ in ice^49,50^ and an average CO_2_ concentration of 300 ppm for the late Holocene^51^, a TIC concentration of 0.0165 mg C L^–1^ is estimated for the contribution of subaqueous meltwater; this value is 2–3 orders of magnitude lower than the [DIC] in streams feeding the MDV lakes^43^. The direct release of atmospheric CO_2_ gases into the water column is not a fractionating process, and the δ^13^C_TIC_ would preserve the δ^13^C of occluded CO_2_ gas (δ^13^C_CO2_ from Dome C, and Talos Dome range from –7.3 to –6.3‰; Refs.^52,53^). Lake Untersee is also recharged by subglacial meltwater, and the DIC in this environment can originate from three sources: melting of basal ice, dissolution of carbonates or oxidation of organics in the bedrock; all three sources have δ^13^C and ^14^C composition that differ substantially. In the Precambrian anorthosite bedrock, the carbonate and organics would be ^14^C dead with δ^13^C values near 0 and c. – 25‰, respectively^54^. This which would result in much lower the F^14^C_TIC_ of the water column and vastly different δ^13^C_TIC_. Therefore, a major contribution from subglacial carbonate dissolution or oxidation of organic carbon (i.e., Refs. ^55,56^) can be ruled out, and the DIC in the subglacial meltwater is likely the result of basal melting with a similar TIC and δ^13^C_TIC_ as the direct melting of the Anuchin Glacier. Dissolution of occluded CO_2_ undergoes hydrolysis to hydrolytic acid (H_2_CO_3_) in the lake, which is then utilized in weathering reactions of plagioclase: the resulting HCO_3_ and thus DIC would have the same δ^13^C and ^14^C composition as the source CO_2_^57^. The DIC/Cl cannot be used to infer the contribution of other sources of DIC to the lake because DIC/Cl in lake is much lower than potential inputs due to significant sequestering of carbon by the mats. The δ^13^C_TIC_ (– 9.1 ± 0.4‰) is slightly lower to the δ^13^C of occluded atmospheric CO_2_ in Antarctic ice cores (– 7.3 to – 6.3‰; Refs.^52,53^) and the minor depletion can be attributed to oxidation of the trace amount of DOC with low δ^13^C in the water column. However, the fact that δ^13^C_TIC_ is not enriched over δ^13^C_CO2_, like most lakes in the MDV, suggest that photosynthesis is not fractionating the DIC reservoir. This can occur in an environment where the phototrophic activity is carbon-starved, and isotope fractionation by cyanobacteria becomes negligible (i.e., Ref.^44^).

**Figure 3:**
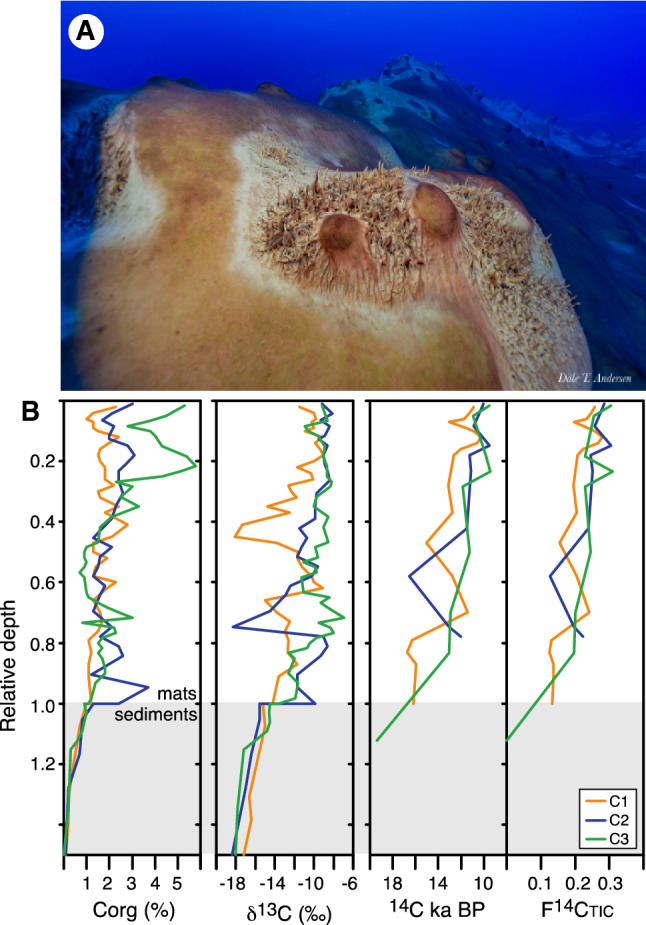
(**A**) Photograph of microbial mats, large conical stromatolites and pinnacles growing in Lake Untersee at 12–13 m depth. Note the clarity of the water column which is a reflection of the nutrient-starved water (e.g., very little DIC or P). (**B**) Organic carbon content, δ^13^C and ^14^C profiles of three microbial mats in Lake Untersee, Antarctica. Cores taken in proximity to photograph shown in A.

The δ^13^C and ^14^C of the microbial mats provide evidence that Lake Untersee is an isotopically indiscriminate carbon-starved ecosystem that utilizes HCO_3_ and CO_2_ sources for phototrophic carbon fixation. The organic C abundances and δ^13^Corg of the microbial mats range from 1.0 to 5.8 wt% and – 6.9 to – 18.1‰, both with a general decrease with depth (Fig. 3). Several mat laminae from the three cores were radiocarbon dated, and the top laminae reported radiocarbon ages of 10,875 ± 37 years BP (Core 1), 10,052 ± 56 years BP (Core 2) and 9,524 ± 48 years BP (Core 3) (Table 4). The bottom mat layers were dated at 16,208 ± 65 years BP (Core 1), 12,031 ± 68 years BP (Core 2) and 13,049 ± 90 years BP (Core 3). Despite some age reversals, a general increase in ^14^C age is observed with depth, indicating that mats are slowly building biomass at a rate of c. 2.5 mm per 100 years. The δ^13^C_TIC_ in lake waters (– 9.1 ± 0.4‰) is similar to the δ^13^C of the top layer of microbial mats (Core 1 = –11.5‰, Core 2 = –9.2‰ and Core 3 = –9.2‰), indicating that little to no ^13^C fractionation is occurring between the DIC pool and the cyanobacteria during carbon fixation. The pH of Lake Untersee is high (~ 10.5) and the dominant DIC species are HCO_3_^–^ and CO_3_^2–^. The cyanobacteria are therefore actively transporting HCO_3_^–^ into their cells where carbonic anhydrase subsequently catalyzes the production of dissolved CO_2_ used for carbon fixation (e.g., Refs.^44,58^). Considering that this process is energetically costly, the cyanobacteria use all of the transferred HCO_3_^-^ and no ^13^C fractionation ensues. The ^14^C results indicate that the cyanobacteria are also fixing respired CO_2_ from heterotrophic metabolism in the underlying microbial mats layers. The top microbial mat layer (10,875 ± 37 years BP to 9,524 ± 48 years BP) are c. 3,000 years older than the TIC pool in the lake waters (^14^C_TIC_ =  ~ 7,000 years BP). Since the mats are accumulating biomass at a rate of c. 2.5 mm per 100 years, the rate of carbon sequestration is not an explanation to the offset. The ^14^C difference between the TIC and top mat layer is attributed to the cyanobacteria fixing carbon from two sources: (1) the HCO_3_^–^ in the water column with δ^13^C_TIC_ of –9.1 ± 0.4‰ and ^14^C_TIC_ near 7,000 years BP, and (2) heterotrophically respired CO_2_ from the decomposition of “older” buried organics with slightly more depleted δ^13^C. Metagenomic analysis for predictive metabolic functions indicates that the heterotrophic communities are capable of metabolic activities typical of recycling of organic material, such as amino–acid metabolism and carbohydrate metabolism^16^.

**Table 4:** Radiocarbon results of microbial mats in Lake Untersee, Antarctica.

Sample ID	Depth (mm)	^14^C year BP	± 2σ	F^14^C	± 2σ	Lab ID
**Core 1 (18.5 m depth)**						
C1-1	2	10,875	37	0.2582	0.0012	UOC-11810
C1-2	4	11,232	38	0.247	0.0012	UOC-11811
C1-3	6	11,731	38	0.2322	0.0011	UOC-11812
C1-4	7	13,072	47	0.1965	0.0011	UOC-11813
C1-5	9	11,541	43	0.2377	0.0013	UOC-11814
C1-6	10	10,921	38	0.2568	0.0012	UOC-11815
C1-7	12	10,222	38	0.2801	0.0013	UOC-11816
C1-8	14	10,569	40	0.2683	0.0013	UOC-11817
C1-9	16	12,045	44	0.2233	0.0012	UOC-11818
C1-10	18	12,617	50	0.2079	0.0013	UOC-11819
C1-15	28	13,119	45	0.1953	0.0011	UOC-11820
C1-19	37	12,737	51	0.2048	0.0013	UOC-11821
C1-22	47	15,034	83	0.1539	0.0016	UOC-11822
C1-26	58	12,751	42	0.2045	0.0011	UOC-11823
C1-30	70	11,437	39	0.2408	0.0012	UOC-11824
C1-33	79	16,307	67	0.1313	0.0011	UOC-11825
C1-34	83	16,729	69	0.1246	0.0011	UOC-11826
C1-35	87	15,950	69	0.1373	0.0012	UOC-11827
C1-36	91	16,032	75	0.1359	0.0013	UOC-11828
C1-37	100	16,208	65	0.133	0.0011	UOC-11829
**Core 2 (13 m depth)**						
C2-1	1	10,052	56	0.2861	0.0020	UOC-9985
C2-5	9	10,882	70	0.2580	0.0022	UOC-9986
C2-8	14	9,510	64	0.3061	0.0024	UOC-9378
C2-9	16	11,268	104	0.2459	0.0032	UOC-9379
C2-12	22	11,113	58	0.2507	0.0018	UOC-9987
C2-19	37	11,481	60	0.2395	0.0018	UOC-9988
C2-24	52	16,567	80	0.1271	0.0012	UOC-9989
C2-29	66	13,040	76	0.1973	0.0018	UOC-9380
C2-30	70	12,031	68	0.2237	0.0018	UOC-9381
**Core 3 (17 m depth)**						
C3-1	1	9,524	48	0.3055	0.0018	UOC-9983
C3-2	3	10,945	82	0.2560	0.0026	UOC-8379
C3-8	11	9,791	58	0.2293	0.0016	UOC-9983
C3-10	14	9,419	76	0.3096	0.0030	UOC-8380
C3-13	17	11,832	58	0.2293	0.0016	UOC-9984
C3-20	30	11,285	80	0.2454	0.0024	UOC-8381
C3-30	42	12,917	78	0.2003	0.0020	UOC-8382
C3-40	53	13,049	90	0.1970	0.0022	UOC-8383
C3-49*	81	19,370	156	0.0009	0.0018	UOC-8384

F^14^C, fraction modern carbon; ^14^C year BP, years before 1950.

*Sediments underlying mats.

## Concluding remarks

Stromatolites may be Earth’s oldest macroscopic fossils and provide plausible evidence of a biosphere on Earth 3.45 GYA^59,60^. Untersee is, at present, the only reported lake hosting the formation of modern, large conical stromatolites morphologically similar to those earliest examples^61^. The lake and its microbial ecosystem provide a glimpse into early Earth and is regarded as an analogue for Enceladus and potential ice-covered water bodies on early Mars^18^. Lake Untersee is recharged by subaqueous melting of glacial ice and subglacial meltwater and plagioclase weathering is a minor contributor of Ca^2+^ and Na^+^. The lake remains ice-covered with no open water along the margin that would provide access to nutrients, CO_2_ and enhanced sunlight^12–14^; yet its floor is colonized by active cyanobacteria forming mats, pinnacles and large conical structures growing in a carbon-starved and light-limited waters^12^. The study highlights the role of carbon cycling within the laminated microbial mats where cyanobacteria utilizes both HCO_3_ and CO_2_ for phototrophic carbon fixation. To survive in this carbon-starved system, there is substantial DIC utilization between the cyanobacteria in the top layer of the mats and the underlying heterotrophs.

## Methods

### Field sampling

Lake water samples were collected at 5–10 m intervals in 2017–2019 using a clean 2.5 L Niskin bottle from holes drilled through the ice cover in the North and South basins. The water samples were immediately transferred into sampling bottles unfiltered due to the sensitivity of the high pH waters to rapid absorption of atmospheric CO_2_ that would affect pH and DIC. Water samples for major and trace ions, were collected in HDPE bottles; waters for TIC, TOC, δ^13^C_TIC_ analyses were collected in 40 mL amber glass vials with a butyl septa cap. Radiocarbon samples were collected in pre-baked 1 L amber glass amber bottles.

Benthic microbial mats along the lake bottom were sampled for organic C content, δ^13^Corg and ^14^C analyses. Three cores were collected near the eastern edge of the push-moraine at depths of 18.5 m (Core 1), 13 m (Core 2) and 17 m (Core 3) by a scientific diver using SCUBA and pushing a 4 mm diameter polycarbonate tube vertically into the mats. Sodium polyacrylate was added to the waters above the mats in the tube to form a gel seal prior to sealing with a rubber stopper. The gel seal preservation method minimizes disturbances to sediment cores during transport and shows no detectable effects on measurements of organic carbon^62^.

### Lake water analyses

Major ions were measured at the Geochemistry Laboratory at the University of Ottawa. Major cations (Na, Ca, Mg, K, Sr) were acidified upon receipt at the university using 2 µL of trace metal grade 10% nitric acid and measured by an Agilent 4,200 inductively coupled plasma atomic emission spectrometer. Major anions (SO_4_, NO_2_, NO_3_, Cl) were measured unacidified using a DIONEX ion-chromatograph. Analytical precision is ± 5% for cations and anions. Charge balance error was < 2.1% when calculated using measured cations-anion and [H^+^] and [OH^–^] for an average pH of 10.6.

Barium sulfate (BaSO_4_) was precipitated from the oxic water samples for stable sulfur isotope (^34^S/^32^S) analysis of total and dissolved sulfate to investigate the source of sulfate in the lake waters. The dried and rinsed precipitates were weighed (~ 0.5 mg) into tin capsules with ~ 1 mg of WO_3_, loaded into the Isotope Cube EA to be flash combusted at 1,800 °C. The released gases were carried by helium through the EA to be cleaned, then separated. The resulting SO_2_ gas was carried into the Delta Plus XP isotope ratio mass spectrometer (ThermoFinnigan, Germany) via a conflo IV interface for ^34^S/^32^S determination. All δ^34^S results are reported as permil deviation relative to VCDT and expressed using the delta-notation. The 2σ analytical precision is ± 0.4‰.

Strontium isotope ratios (^86^Sr/^87^Sr) were measured in lake waters to investigate potential sources of weathering. The Sr isotope ratios were measured at Queen’s University Facility for Isotope Research (QFIR) using a ThermoFinnigan Neptune MC–ICP–MS with all ratios normalized to ^86^Sr/^88^Sr ratio of 0.1194 to account for mass fractionation. Results are reported as the mean of 63 consecutive measurements and corrected to internally normalized NIST standard NBS 987 (^87^Sr/^86^Sr = 0.710291). Reported error (2σ) is ± 0.0002 to 0.005. The replicate samples were within the analytical uncertainty.

Tritium (^3^H) was measured by electrolytic enrichment and liquid scintillation counting. Samples were pretreated prior to enrichment by de-ionizing the waters using an ion-exchange resin and shaken vigorously for 4 h. The deionized waters were mixed with sodium peroxide (Na_2_O_2_) and electrolytically enriched in metal cells. Samples were enriched at ~ 5.8 amps for 5 days, reducing the volume from 250 mL to approximately 13 mL. The enriched samples were then decay counted on a low-background Quantilus liquid scintillation counter. Results are reported in tritium units (TU), where1 TU = 0.11919 Bq/L; 2σ analytical precision is 0.8 TU.

Radiodine (^129^I) was extracted from aqueous samples using a NaI carrier-addition extraction method and precipitated as AgI, using methods described in Marsh (2018). Targets were prepared by mixing 1–2 mg of AgI with high–purity niobium powder, and pneumatically pressed into a stainless–steel cathode for analysis of ^129^I by AMS (HVE 3MV Tandetron). Measurements were normalized to the ISO–6II reference material by calibration with the NIST 3,230 I and II standards. Results are reported as the measured ^129^I/^127^I ratio 10^–14^ (with carrier), the calculated ratio (without carrier) and the calculated sample ^129^I concentration (atoms/L). The ^129^I concentrations without carrier are calculated based on total iodine measured by ICP–MS, amount of I-carrier added, and the AMS measured ^129^I/^127^I ratio.

The TIC-TOC concentration and their stable isotope ratios (δ^13^C_TIC_, δ^13^C_TOC_) in lake waters were measured by a wet TOC analyzer interfaced with a Thermo DeltaPlus XP isotope-ratio mass spectrometer using methods described by Ref.^63^ at the Ján Veizer Stable Isotope Laboratory, University Ottawa. The isotope ratios are presented as permil deviation relative to VPDB and expressed using the delta-notation. The 2σ analytical precision is ± 0.5 ppm for TOC and TIC concentrations and ± 0.2‰ for δ^13^C_TIC_ and δ^13^C_TOC_. The detection limit of TIC and TOC is < 0.3 ppm.

Radiocarbon analysis of waters was performed at the A.E. Lalonde Accelerator Mass Spectrometry Laboratory, University of Ottawa. Sample preparation, extraction of inorganic and organic from waters and graphitization is described by Murseli et al.^64^. Graphitized samples were analyzed on a 3MV tandem mass spectrometer and the ^14^C/^12^C ratios are expressed as fraction of Modern Carbon (F^14^C) and corrected for spectrometer and preparation fractionation using the AMS measured ^13^C/^12^C ratio^65^. Radiocarbon ages are calculated as − 8033ln(F^14^C) and reported in ^14^C year BP (BP = AD1950) as described by^66^. The 2σ errors are reported with the results below and are below 0.013 F^14^C (or < 190 years).

### Microbial mat analyses

The three microbial mat cores were first extruded from the polycarbonate tube onto a clean surface, and each core was divided in half lengthwise using a stainless-steel spatula. The core material consisted of 6–10 cm thick laminated microbial mats that transitioned sharply to sediments. The mat laminae were 0.2–1 mm thick and composed of organic material intermixed with silt and clay-sized particles. In the uppermost section of the cores, the microbial mat laminae were peeled individually, and sampling resolution increased to several laminae (1–10 mm resolution) before the mats transitioned to the underlying sediments which were sampled at 0.5–2 cm resolution. All samples were dried in aluminum trays at 55 °C, weighed, crushed to a powder using a mortar and pestle and acidified with 10% HCl to remove inorganic carbonates.

The mineralogy of sediments in the mats was determined on powdered samples using a Rigaku PXRD using a high intensity 10 mm slit and a scan speed of 0.2° per minute for optimal peak resolution. The resulting intensities were peak matched to mineral phases known to exist in the region and Rietveld Refinement was done to provide relative phase abundances.

Samples were analyzed for organic C content and δ^13^C_org_ a by purge and trap chromatography on an elemental analyzer (Vario Isotope Cube, Elementar) coupled via a Conflo III interface to a Thermo DeltaPlus isotope ratio mass spectrometer. All samples were weighed in tin capsules, mixed with tungsten oxide and flash combusted at 1,800 °C with the resulting CO_2_ gases measured separately by the thermal conductivity detector. The δ^13^C values are reported against VPDB using internal standards calibrated to international standards. The analytical precision of organic C is ± 0.1% and for δ^13^C is ± 0.2‰. Results from duplicate samples were within the analytical uncertainty.

Sub-samples of individual laminae of microbial mats in the three cores were submitted to the AMS laboratory for ^14^C analysis where the samples were pretreated (e.g., freeze-dried, acid-treated to remove inorganic C) according to methods described by Ref.^65^. For organic C extraction, the pretreated and dried sediments were combusted using a Thermo Flash 1,112 elemental analyzer (EA) in CN mode interfaced with an extraction line to remove non-condensable gases and trap the pure CO_2_ in a prebaked 6 mm Pyrex breakseal^65^. The CO_2_ in the breakseal was graphitized, measured, background corrected and reported using the same methods described for the lake water samples.

### Description of ice core datasets

Lake Untersee is recharged by glacial meltwater (subaqueous melting of the Anuchin Glacier and subglacial meltwater), however deep ice core from the Anuchin Glacier and nearby ice field do not exist. To gain insight into solutes content from the glacial meltwater, the chemistry of Lake Untersee is compared to the following ice core datasets.

*Western Dronning Maud Land.*

CM cores (CM). 360 m a.s.l., 140 km from coast, 30–300 years, δ^34^S_tot_. Ref.^31^.

Core Epica. 700 m a.s.l., 200 km from the coast, 1865–1991. Ionic content. Ref.^28^.

Core Victoria (CV). 2,400 m a.s.l., 550 km from the coast, 30–1,100 years, δ^34^S_tot_. Ref.^31^.

*Dome C*. 3,240 m a.s.l., Holocene and late Pleistocene. Ionic content and δ^34^S_tot_. Refs.^34,34^.

*Taylor Dome*. 2050 m a.s.l. Holocene and late Pleistocene. Ionic content. Ref.^35^.

*Vostok*. 3,420 m a.s.l., Holocene and late Pleistocene, δ^34^S_tot_. Ref.^36^.
